# The Intramolecular Self‐Assembly of Statistical Copolymers in Aqueous Solution to Form Anisotropic Single‐Chain Nanoparticles with Tunable Aspect Ratio

**DOI:** 10.1002/marc.202400898

**Published:** 2024-12-27

**Authors:** Thomas J. Neal, Rebecca E. Stone, Csilla György, Svetomir B. Tzokov, Sebastian G. Spain, Oleksandr O. Mykhaylyk

**Affiliations:** ^1^ School of Mathematical and Physical Sciences University of Sheffield Dainton Building Sheffield S3 7HF UK; ^2^ ICON 500 South Oak Way, Green Park Reading RG2 6AG UK; ^3^ School of Biosciences University of Sheffield Firth Court, Western Bank Sheffield S10 2TN UK

**Keywords:** amphiphilic copolymers, proteins, SAXS, self‐assembly, single‐chain nanoparticle

## Abstract

Natural single‐chain nanoparticles (SCNPs) such as proteins have inspired research into the formation and application of synthetic SCNPs. Although the latter can mimic general aspects of the self‐assembly behavior of their biological counterparts, these systems remain relatively understudied. In this respect, a systematic series of amphiphilic statistical copolymers (ASC) of different molecular weights, with a hydrophilic comonomer (methacrylic acid) and varying hydrophobic comonomer to encompass methacrylates of different hydrophobicity, are synthesized. Small‐angle X‐ray scattering studies confirmed that SCNPs are achieved for each copolymer series when dispersed in basified water at 1% w/w. When the aggregation number of the ASC nanoparticles is close to unity the particle shape elongates resulting in a larger particle surface area to volume ratio, allowing more hydrophilic groups to locate on the particle surface tending to keep the particle surface charge density (PSC) constant. Thus, within a series, particle elongation increases with copolymer molecular weight. Structural parameters of SCNPs formed by ASCs composed of hydrophobic components with low partition coefficients are well consistent with predictions obtained from the PSC model. These results highlight the main parameters, namely molecular weight and acid content, responsible for the SCNP formation and provide insight into how specific particle morphology can be targeted.

## Graphical abstract

The facile synthesis of amphiphilic statistical copolymers that fold into single‐chain nanoparticles (SCNP) with an emerging anisotropic shape, a prominent feature of proteins, is presented. The morphology of these synthetic SCNPs is controlled by the polymer molecular weight and composition. Increasing the copolymer molecular weight induces particle elongation.

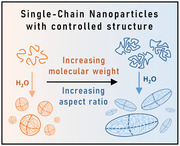

## 1 Introduction

Over the past few years, the topic of single‐chain nanoparticles (SCNPs) has become increasingly popular with polymer scientists and the wider scientific community.^[^ ^1^, ^2^, ^3^, ^4^, ^5^, ^6^, ^7^, ^8^, ^9^, ^10^, ^11^, ^12^, ^13^, ^14^, ^15^, ^16^, ^17^, ^18^ ^]^ The growing interest surrounding these materials stems from the similarities they share with many biomolecules such as proteins.^[^ ^9^ ^]^ Like most proteins, SCNPs are composed of a single chain that, depending on intra‐molecular interactions and the surrounding medium, folds and packs itself into a certain shape to achieve the lowest energy state. Through the folding mechanism of self‐assembly, both proteins and SCNPs create small functional areas within the larger particulate structure. Inspired by the utility of these similar biomolecules, SCNPs have been used as catalytic nanoreactors,^[^ ^1^, ^19^, ^20^, ^21^, ^22^, ^23^, ^24^ ^]^ sensors,^[^ ^25^, ^26^ ^]^ and drug delivery systems.^[^ ^27^ ^]^

In addition to adding functionality to SCNPs, a lot of research has focused on investigating different mechanisms for inducing chain folding. Often, chain folding is achieved through intrachain cross‐linking including covalent^[^ ^28^ ^]^ (e.g., via cycloaddition reactions^[^ ^29^, ^30^, ^31^ ^]^ and photodimerization^[^ ^32^ ^]^), dynamic covalent^[^ ^26^ ^]^ (e.g., hydrazones^[^ ^25^ ^]^ and disulfides)^[^ ^33^ ^]^ and non‐covalent (e.g., hydrogen bonding^[^ ^16^, ^34^, ^35^ ^]^ and metal ligation)^[^ ^11^ ^]^ bonding. It was also demonstrated that hydrophobic interactions on their own could lead to chain folding and the formation of SCNPs in an aqueous environment.^[^ ^2^ ^]^ In this work, amphiphilic statistical methacrylate copolymers with poly(ethylene glycol) (PEG) and alkyl pendant groups were synthesized and dispersed in water. The copolymer chains folded and collapsed through the hydrophobic interactions between the pendent alkyl groups while the hydrophilic PEG groups conferred colloidal stability in the aqueous environment.^[^ ^2^ ^]^ However, despite demonstrating that SCNPs were formed of an arbitrary size, the particle shape was not investigated.

In analogy to biological objects formed by single polymer chains such as proteins, there are two important questions that could be raised: 1) why do SCNPs form, and 2) what is the resultant particle shape and structure? Since SCNPs are composed of a single polymer molecule, commonly having a size of a few nanometers, these questions can be addressed by using X‐ray/neutron scattering techniques and/or high‐resolution microscopy enabling structures at such small length scales to be investigated.^[^ ^5^ ^]^ It has recently been found by X‐ray scattering measurements on amphiphilic statistical copolymers (ASCs) of alkyl methacrylates and methacrylic acid dispersions in water that the SCNP formation depends on the molecular acid (stabilizing unit) content suggesting that the key factors responsible for the particle formation are polymer molecular weight and content of the solvophilic component.^[^ ^36^ ^]^ This aligns with other studies showing that the primary structure of random copolymers, including their composition and chain length, can effectively be used to control the aggregation number of self‐assembled molecules and particle size.^[^ ^37^, ^38^ ^]^ Using a combination of transmission electron microscopy (TEM) and X‐ray scattering it was shown that amphiphilic statistical copolymers form spherical SCNPs at particular compositions and molecular weights.^[^ ^13^ ^]^ Furthermore, it was demonstrated that amphiphilic multiblock statistical copolymers form more complex multi‐compartment structures.^[^ ^12^, ^13^ ^]^ The shape formation of SCNPs was also investigated in polymers where chain folding was induced by hydrogen bonding between benzene‐1,3,4‐tricarboxamide (BTA) groups.^[^ ^16^ ^]^ It was found that the hydrogen bonding between BTA groups led to the formation of an interior helical structure resulting in elongated particles. In analogy to proteins, these studies demonstrate how different copolymers self‐fold to form various structures of SCNPs. However, the factors responsible for causing these particles to form a particular shape and the underlying principles behind this phenomenon have yet to be determined. Herein, a series of ASCs comprised of methacrylic acid (MAA) and a range of alkyl methacrylates was synthesized using reversible addition‐fragmentation chain transfer (RAFT) polymerization. These copolymers were subsequently dispersed in water and their structures were analyzed using small‐angle X‐ray scattering (SAXS). It was found that the degree of polymerization (DP) of the ASC affected the SCNP formation as well as the particle morphology with a transition from spherical to ellipsoidal (elongated) with increasing DP and that higher DP polymers formed dispersions of elongated particles with higher aspect ratios. This work demonstrates the clear effect that DP and polymer chemical composition, controlling a balance of hydrophobic and hydrophilic components, have on the SCNP morphology, allowing particle anisotropy, a prominent feature of proteins, to be achieved. The obtained results give further insight into the formation of nanoparticles by single polymer molecules and provide a valuable understanding of the driving forces leading to this structural assembly mimicking biological systems.

## 2 Results and Discussion

### 2.1 Synthesis of Amphiphilic Statistical Copolymers by RAFT Solution Polymerization

**Figure 1:**
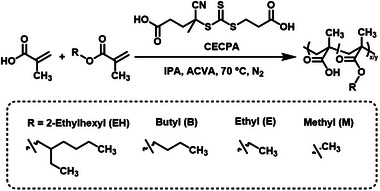
RAFT solution polymerization of MAA with either EHMA, BMA, EMA, or MMA to form P(EHMA_y_‐*stat*‐MAA_x_), P(BMA_y_‐*stat*‐MAA_x_), P(EMA_y_‐*stat*‐MAA_x_) and P(MMA_y_‐*stat*‐MAA_x_), respectively. The copolymerization of MAA with either EHMA, BMA, EMA or MMA was performed in IPA at 50% w/w.

A series of amphiphilic alkyl methacrylate‐based statistical copolymers was synthesized in which both the hydrophobic comonomer and the degree of polymerization were systemically varied (**Figure** **1**). In all cases, the hydrophilic comonomer was methacrylic acid (MAA) while the hydrophobic comonomer was varied to encompass methacrylates of different hydrophobicity. 2‐ethylhexyl (EH), *n‐*butyl (B), ethyl (E), and methyl (M) methacrylate (MA) (abbreviated as *X*MA, where *X* represents a particular methacrylate) were examined. P(*X*MA*_n_*‐*stat*‐MAA*_m_*) copolymers, where *n* and *m* are the mol% of *X*MA and MAA units in the comonomer feed, respectively, were synthesized via RAFT solution copolymerization in isopropanol (IPA) at 50% w/w (Figure 1).

Previously, it was shown that the size of particles formed by self‐assembly of ASCs is independent of degree of polymerization (and thereby molecular weight) with the aggregation number of the particle increasing as the molecular weight of the copolymer is reduced.^[^ ^39^ ^]^ However, a critical monomer composition (critical hydrophile content), specific to each monomer pair, was also observed where this independence no longer holds true as the polymers form SCNPs.^[^ ^36^, ^39^ ^]^ As SCNPs contain a single chain, it can be expected that their volume should increase with increasing DP of the polymer as the length of a single chain increases. With this in mind, a series of copolymers was synthesized for each monomer pair with targeted DPs of 100, 250, 500, and 1000. The *X*MA:MAA composition ratio targeted for the synthesized statistical copolymers containing methyl methacrylate (MM), ethyl methacrylate (EM), *n‐*butyl methacrylate (BM), and 2‐ethylhexyl methacrylate (EHM) were 80:20, 70:30, 50:50, 35:65, respectively, with the aim of forming SCNPs (**Table** **1**). These compositions were chosen based on previous work^[^ ^36^ ^]^ and an initial screening of different compositions using a single DP of 250.

**Table 1:** Molecular composition and molecular weights of synthesized poly(alkyl methacrylate‐stat‐methacrylic acid) [P(*X*MA‐*stat*‐MAA)] copolymers measured by GPC calibrated against near‐monodisperse PMMA standards using a THF and acetic acid (4% v/v) mobile phase.

*X*MA	Copolymer name	Targeted DP (*M*_n_^target^, kDa)	Targeted Composition (*X*MA:MAA)	GPC		
				*M*_n_, kDa	*M*_w_, kDa	*M*_w_/*M*_n_
MMA	MM_100_	100 (9.6)	70:30	11.1	12.7	1.15
	MM_250_	250 (24.0)	70:30	22.1	25.9	1.17
	MM_500_	500 (47.9)	70:30	40.5	48.5	1.20
	MM_1000_	1000 (95.8)	70:30	63.0	77.6	1.23
EMA	EM_100_	100 (10.3)	60:40	11.8	13.5	1.14
	EM_250_	250 (25.7)	60:40	28.2	32.4	1.15
	EM_500_	500 (51.4)	60:40	48.8	59.1	1.21
	EM_1000_	1000 (102.8)	60:40	105.6	137.3	1.30
BMA	BM_100_	100 (11.4)	50:50	12.8	14.6	1.14
	BM_250_	250 (28.5)	50:50	30.4	35.2	1.16
	BM_500_	500 (57.0)	50:50	59.5	70.3	1.18
	BM_1000_	1000 (114.0)	50:50	102.1	134.5	1.32
EHMA	EHM_100_	100 (12.5)	35:65	14.6	16.6	1.13
	EHM_250_	250 (31.3)	35:65	30.0	34.6	1.15
	EHM_500_	500 (62.7)	35:65	52.2	63.6	1.22
	EHM_1000_	1000 (125.4)	35:65	86.4	103.4	1.30

**Figure 2:**
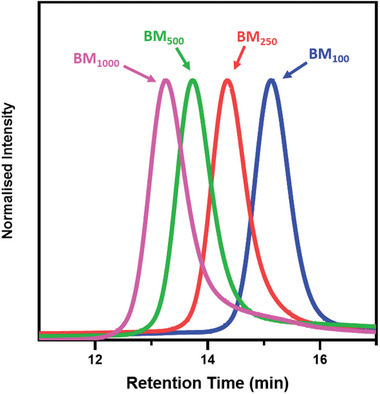
GPC chromatograms of the P(BMA‐*stat*‐MAA) copolymer series synthesized by RAFT solution polymerization at 50% w/w in IPA. GPC was performed in THF containing 4% v/v acetic acid against PMMA standards.

It was observed previously that synthesizing P(BMA‐*stat*‐MAA) in IPA at 20% w/w led to poor conversion of the MAA monomer.^[^ ^39^ ^]^ However, performing the copolymerization at 50% w/w was found to help both the monomers react at a similar rate and reach high conversions.^[^ ^36^ ^]^ Kinetic studies of the polymerizations show that this remains the case for all studied systems (Figures S1 and S2, Supporting Information). Since both monomers used for the ASC syntheses reach high conversions (> 99%, Figure S1, Supporting Information) and react at similar rates (Figure S2, Supporting Information), the distribution of the hydrophobic and hydrophilic along the copolymer backbone can be described as statistical rather than blocky. The EMA:MAA system showed the largest difference in polymerization rates; however, the consumption of each monomer remains similar enough to result in a largely statistical distribution of each monomer along the copolymer backbone. Furthermore, due to the near‐to‐complete conversion achieved by both monomers during the synthesis of these copolymers (Figure S1, Supporting Information), the final copolymer compositions were assumed to be close to those targeted (Table 1). Molecular weights were determined by GPC against poly(methyl methacrylate) (PMMA) standards (Table 1 and **Figure** **2**). In all cases, the *M*_n_ increases with the targeted DP although there are slight deviations from the targeted values. Some deviations are expected due to differences in the hydrodynamic volume of the copolymers compared to the PMMA standards. Additionally, deviations may result from interactions between the copolymer's acidic groups and the GPC column, especially for higher molecular weight copolymers. Although acetic acid was added to the mobile phase to minimize these interactions, some residual effects may persist. The majority of the copolymers have narrow molecular weight distributions (*M*_w_/*M*_n_ < 1.22, see Table 1 and Figure 2) showing that the RAFT copolymerization is well controlled. Some loss of control is evident (*M*_w_/*M*_n_ ~ 1.3) when a DP of 1000 was targeted in the syntheses. This loss of control within the RAFT synthesis is expected as the molecular weight of the copolymer is increased.^[^ ^40^ ^]^ The GPC chromatograms show that the distributions are generally monomodal and no high molecular weight shoulders (often as a consequence of termination by combination or disulfide formation from hydrolyzed RAFT end groups) were observed (Figures 2 and S3, Supporting Information).

### 2.2 Aggregation Behavior of Amphiphilic P(*X*MA‐*stat*‐MAA) Statistical Copolymers

Aqueous copolymer dispersions were obtained using a solvent switch method from a good solvent (IPA).^[^ ^39^ ^]^ Since these copolymers were synthesized in IPA at a relatively high concentration (50% w/w) and the monomer conversion was found to be near 100%, the amount of any residual monomer in the product will be negligible. Thus, these solutions were dispersed in water without further purification. The copolymer solutions (50% w/w in IPA) were diluted slowly with water in the presence of triethanolamine (TEA, 1 molar equivalent relative to the MAA residues). This organic base was added to the solution to deprotonate the MAA units and induce a negative charge to stabilize the dispersions. These samples were diluted further to 1% w/w to reduce the inter‐particle interactions that are present at high copolymer concentrations.

**Figure 3:**
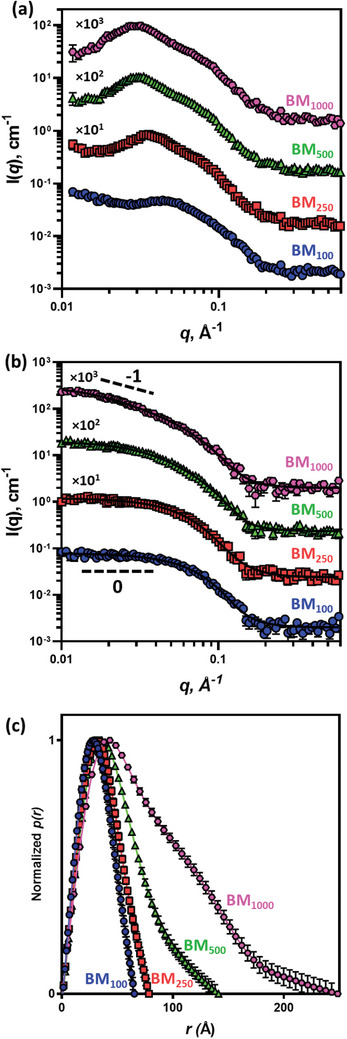
a) SAXS patterns recorded for 1.0% w/w aqueous dispersions of P(BMA‐*stat*‐MAA) copolymer particles (symbols) and b) SAXS patterns recorded for 1.0% w/w aqueous dispersions of P(BMA‐*stat*‐MAA) copolymer particles with 0.05 m NaCl (symbols) fitted using an ellipsoid core–shell model with a linear background [Equations (S2–S8,S13, Supporting Information)] (solid lines). The dashed guiding lines show a gradient of scattered intensity. A Xenocs Xeuss 2.0 SAXS instrument was used for these measurements. Some patterns are shifted upward by an arbitrary numerical factor (shown next to each pattern) to aid clarity, and c) the pair‐distance distribution functions, *p*(*r*), obtained from experimental SAXS patterns of BM_100_, BM_250_, BM_500_, and BM_1000_ (see Figure 3b) using regularization technique.^[^ ^42^ ^]^ All *p*(*r*) functions are normalized to their own maximum.

SAXS was performed on the 1% w/w aqueous copolymer dispersions to assess the copolymer morphology in solution (**Figure** **3**a). SAXS was used here as it is considered far more statistically robust than TEM, with the latter technique also prone to staining artifacts and the possibility of nano‐object flattening occurring during drying. Furthermore, TEM is not an ideal structural analysis method for this study as the particle dimensions are often below the reliable resolution of the microscope.^[^ ^36^, ^39^ ^]^ Despite the measurements being performed on a relatively low concentration of dispersions, these scattering patterns display a structure factor peak at low *q* (*q* < 0.05 Å^−1^, Figure 3a). As indicated by previous electrophoretic measurements, performed on a similar statistical amphiphilic copolymer system, the particles are charged and the structure factor peak is expected due to inter‐particle electrostatic repulsion.^[^ ^39^ ^]^ Considering that multi‐molecular assemblies of similar statistical copolymers form spherical particles,^[^ ^36^ ^]^ a model describing scattering intensity from spherical‐shaped particles was initially chosen to fit the collected SAXS data. A SAXS model based on spherical core‐shell form factor (to account for both the copolymer particle and the associated cation shell,^[^ ^36^ ^]^ Equations (S2–S9, Supporting Information) when the aspect ratio is set to 1) with the Hayter‐Penfold mean spherical approximation (HP) structure factor [to account for the interparticle interactions of charged particles, Equation (S15, Supporting Information)] and a linear background (to account for the plateau in intensity in high *q* likely to be caused by fluctuations in scattering length density across the particle) [Equation (S14, Supporting Information)] was fitted to these scattering patterns. However, this model in some cases yielded unrealistic results (e.g., the mean particle radius was smaller than its standard deviation) suggesting that the spherical particle shape chosen for the SAXS model might not be always correct.

In order to identify the particle shape, it is desirable to obtain scattering patterns representing the particle form factor only. Consequently, the presence of the structure factor peak complicates the determination of the particle morphology. A small amount of NaCl (50 mm) was added to the 1% w/w dispersions to partially shield the particle charge while keeping the particle dispersions stable. This approach enabled the structure factor peak to be removed from the scattering patterns^[^ ^39^ ^]^ (Figure 3b).

The SAXS pattern of the BM_100_ dispersion with added salt has a near zero gradient for the scattered intensity in the low *q* region (*q* < 0.05 Å^−1^) suggesting a spherical shape of the particles (Figure 3b). However, upon increasing the copolymer DP the scattered intensity gradient is reduced and approaches −1 (Figure 3b, compare SAXS patterns for BM_100_ and BM_1000_). A slope of −1 in the low *q* (Guinier) region is characteristic of rod‐like particles so this observation indicates that the formed particles may elongate as the DP increases.^[^ ^41^ ^]^

The observation of elongated particles was further confirmed using pair‐distance distribution function (PDDF) analysis (Figure 3c).^[^ ^42^ ^]^ In this analysis, the PDDF, *p*(*r*), for BM_100_ shows a relatively symmetrical distribution of distances that could be assigned to polydisperse spherical particles. However, as the copolymer DP increases the PDDFs evolve into a shape characteristic for elongated (prolate ellipsoid or rod‐like) particles.^[^ ^41^ ^]^ The formation of an elongated structural morphology (multi‐core flower necklace) above a critical DP has been previously reported for amphiphilic copolymer systems.^[^ ^43^ ^]^ However, this morphology was associated with a highly ordered, alternating sequence of amphiphilic blocks within the copolymers, with large core‐forming pendant groups. This is in stark contrast to the statistical copolymers discussed here, which possess a significantly different molecular architecture, favoring a gradual elongation with increasing DP.

Following the preliminary results of the particle shape analysis (Figure 3b,c) and the fact that PMAA‐based statistical copolymers form a core–shell particle morphology,^[^ ^36^ ^]^ an ellipsoid (prolate) core–shell model, counting the particle elongation, [Equations (S2–S8,S13, Supporting Information)] was fitted to the experimental

SAXS patterns. The model fitted well with the experimental scattering patterns of the BM dispersions (Figure 3b) and those recorded for the other copolymer dispersions (Figure S4, Supporting Information). To further confirm the formation of elongated particles, cryogenic electron microscopy (cryo‐EM) images of the EHM_100_ and EM_1000_ dispersions were collected. Due to the small particle size, high resolution is required, and imaging via TEM is extremely difficult. Nevertheless, spherical particles were identified for the EHM_100_ dispersion, whereas the EM_1000_ dispersion showed particles that were elongated (Figure S5, Supporting Information) in good agreement with SAXS results (**Table** **2**).

**Table 2:** Structural characteristics of 1.0% w/w poly(alkyl methacrylate‐stat‐methacrylic acid) [P(*X*MA‐*stat*‐MAA)] copolymer dispersions obtained from SAXS analysis: the mean equatorial particle radius (*R*_e_), the polar particle radius (*R*_p_), the mean aggregation number (*N*_agg_) as calculated using Equation (S10, Supporting Information), and the particle aspect ratio (ε = *R*_p_/*R*e). *The table rows in italic and bold font show threshold parameters predicted from PSC model^[^ ^36^ ^]^ for the formation of SCNP by the studied copolymer compositions: MMA:MAA = 70:30; EMA:MAA = 60:40; BMA:MAA = 50:50; and EHMA:MAA = 35:65 (see Supporting Information for details).

*X*MA	Copolymer name	Targeted DP	*R*_e_ [Å]	*R*_p_ [Å]	*ε*	*N*_agg_
MMA	*MM_14_	*14*	*7.6*	*7.6*	*1.0*	*1*
	MM_100_	100	12	23	1.9	1
	MM_250_	250	12	44	3.7	1
	MM_500_	500	13	94	7.2	1
	MM_1000_	1000	14	122	8.7	1
EMA	*EM_43_	*43*	*11.6*	*11.6*	*1.0*	*1*
	EM_100_	100	10	40	4.0	1
	EM_250_	250	13	73	5.6	1
	EM_500_	500	13	109	8.4	1
	EM_1000_	1000	18	169	9.4	1
BMA	BM_100_	100	17	39	2.3	3
	*BM_179_	*179*	*19.4*	*19.4*	*1.0*	*1*
	BM_250_	250	19	49	2.6	2
	BM_500_	500	20	82	4.1	2
	BM_1000_	1000	20	152	7.6	1
EHMA	EHM_100_	100	18	18	1.0	2
	EHM_250_	250	25	25	1.0	2
	EHM_500_	500	21	63	3.0	2
	EHM_1000_	1000	24	72	3.3	1
	*EHM_1108_	*1108*	*37.5*	*37.5*	*1.0*	*1*

While salt ions screen the interparticle interactions, they may be expected to affect the particle size and shape of the dispersions.^[^ ^44^ ^]^ To determine if this was the case, additional experiments studying the dispersion behavior at different salt concentrations were performed. Two copolymer dispersions that formed either spherical particles or elongated particles (EHM_250_ and BM_500_, respectively, see Table 2) were selected for these experiments. The salt concentration was varied systematically from 0 mm to 150 mm and the copolymer dispersions were investigated using SAXS (Figures S6 and S7, Supporting Information). The subsequent SAXS patterns were analyzed using an ellipsoid (prolate) core–shell model with an integrated HP structure factor [Equations (S2–S8,S13,S14, Supporting Information)]. Application of the HP mean spherical approximation for a structure factor may not be completely valid for describing the interactions between charged ellipsoidal particles formed by BM_500_,^[^ ^45^ ^]^ but this approach is justified for the interactions between charged spherical particles of EHM_250_. The addition of salt has an immediate effect on the structure as the salt shields the particle charges and reduces the interaction between particles indicated by the diminishing structure factor peak at *q* = 0.04 Å^−1^ (Figures S5 and S6 (Supporting Information), SAXS pattern corresponding to 12.5 mm of NaCl). The SAXS analysis determined the effective volume fraction (*f*_HP_) to be zero at 50 mm NaCl suggesting that the structure factor peak is completely removed by this salt concentration. These results (Figures S6b and S7b, Supporting Information) show that the particle size and shape virtually do not change across the entire range of salt concentrations and demonstrate that the addition of salt mainly affects the particle‐particle interaction and not the particle morphology. Thus, the presented set of experiments shows only a minor (if any) effect of NaCl on the particle dimensions and shape and, subsequently, validates the approach used in this study for diminishing the interaction of charged particles to simplify the SAXS analysis.

The SAXS data analysis shows that the copolymers self‐assemble into nanoparticles that elongate (the particle aspect ratio, *ε*, grows) with increasing copolymer DP (Table 2). It was previously demonstrated that when several amphiphilic statistical copolymers assemble, they form a spherical particle where its radius is dependent on the copolymer composition but not on the copolymer molecular weight.^[^ ^39^ ^]^ This behavior can be successfully described by the particle surface charge density (PSC) model where the particle radius is related to the fraction of hydrophile (MAA) units occupying the particle surface.^[^ ^36^, ^39^ ^]^ However, results of the current work show that when the aggregation number approaches 1, the copolymer molecular weight has a clear effect on the particle shape and size, with heavier (longer) copolymer molecules forming elongated particles (Table 2).

**Figure 4:**
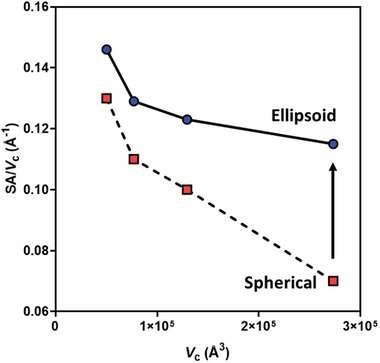
The particle surface area per particle volume (SA/*V*_c_) against the particle volume (*V*_c_) for the BM copolymer ellipsoid particles calculated from SAXS results (circles) and the hypothetical spherical particles calculated based upon the known volumes of the ellipsoid particles (squares). The lines are shown for eye guidance.

The phenomenon by which the particles, including SCNPs, elongate can be rationalized by the inherent behavior of amphiphilic copolymers and the minimization of energy to achieve the most favorable position for both the hydrophilic and hydrophobic groups. In an aqueous dispersion of amphiphilic copolymer particles, the hydrophilic groups locate preferentially on the particle surface to increase favorable interactions with the aqueous phase, whereas the hydrophobic groups will locate preferentially in the core of the particle to minimize these interactions. When a large number of ASCs form a particle, the particle is spherical and its radius is related to the hydrophile component content via the particle surface area, which is described mathematically using the PSC model.^[^ ^36^ ^]^ Mobility of the hydrophile component provided by a large number of molecules enables the molecules to form the most energetically favorable spherical shape. However, when the molecule aggregation number approaches unity, the hydrophile mobility confined by the molecules should come into play. Hypothetically, if the particle shape remains spherical as the particle volume increases with the copolymer DP, then the surface area to volume ratio of the particle [SA/*V*_c_, where SA is calculated using Equation (S16, Supporting Information)] reduces significantly. Consequently, upon increase of the copolymer DP (or molecular weight) at a fixed ratio of hydrophobic and hydrophilic units, more hydrophilic groups would be deepened in the particle core thereby decreasing their number per unit surface area of the particles resulting in a reduction of copolymer favorable interactions with the aqueous phase stabilizing the particle (**Figures** **4** and S8, Supporting Information, dashed lines). However, if the particle elongates as the particle volume increases then the reduction in SA/*V*_c_ is lessened and tends to plateau at a certain value (Figures 4 and S8, Supporting Information, solid lines), likely to be defined by the molecule composition. This means that fewer hydrophilic groups are forced into the bulk of the particle and a consistent amount of them are able to locate on the particle surface.

**Figure 5:**
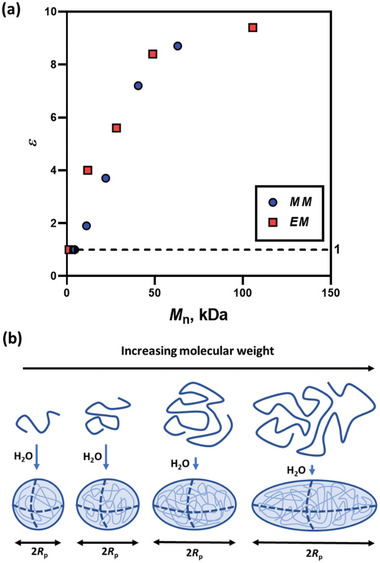
The statistical copolymer SCNP elongation with respect to the copolymer molecular weight: a) experimental data obtained for MMA:MAA = 70:30 (black circles) and EMA:MAA = 60:40 (grey squares) copolymer series, two data points with *ε* = 1 (indicated by the dashed line and the half shaded symbols) corresponds to threshold conditions for the formation of SCNP predicted by the PSC model (see Table 2 and the Supporting Information); and b) schematic showing the particle elongation, where Rp is the polar particle core radius.

The ASCs composed of alkyl methacrylate comonomer with less hydrophobic character (lower partition coefficient, log*P*), such as MM and EM,^[^ ^36^, ^39^ ^]^ tend to form SCNPs which are elongated upon the increase of DP (Table 2). For example, in the MM series, *ε* increases from 1.9 to 8.7 as the DP is increased from 100 to 1000, revealing that elongated (ellipsoidal) particles form at certain molecular weights (**Figure** **5**). Moreover, these results are consistent with the estimations of minimum molecular weight (or DP) required for the formation of spherical SCNPs for this ASC composition (Figure 5a and Table 2), calculated using the PSC model (see Table S1, Supporting Information and associated text in Supporting Information). The calculated points and the experimental points for both the MM and EM copolymer series lie on the same trend curve (Figure 5a). This observation suggests that the SCNP elongation is mainly controlled by the ASC molecular weight. When the molecular volume is close to the critical spherical particle volume, dictated by the ASC composition,^[^ ^36^, ^39^ ^]^ SCNPs are formed. Thus, upon the DP increase, when the statistical copolymer exceeds the critical volume, particle elongation takes place (Figure 5b).

However, it should be noted that the ASCs containing comonomers with higher log*P*, such as BM and EHM, demonstrate particle elongation before the *N*_agg_ reaches 1 (Table 2). The elongation occurs when the minimum molecular weight (or DP) required for the formation of spherical SCNPs for this ASC composition, predicted by the PSC model, has not been reached yet (Table 2). This observation suggests that the main underlying mechanism of the elongation, dictated by the ASC molecular weight, is influenced by additional factors. The “early‐elongation” observed for the BM and EHM series could be caused by the high MAA content present in these copolymers facilitating distortion (elongation) of the particles from spherical shape when *N*_agg_ is close but more than 1. However, these additional factors influencing the SCNP particle shape formation would require further investigation. Nevertheless, the molecular compositions explored in this work, and the results obtained, offer valuable insights into how SCNPs assemble, enabling specific structural morphologies to be targeted and formulated. Furthermore, the behaviors and dependencies observed for the studied, relatively simple, model synthetic systems with a variable acid group content have the potential to further the understanding of more complex and intricate self‐assemblies found in the natural world.

## 3 Conclusion

In order to study the effect of amphiphilic statistical copolymer composition and molecular weight on the single‐chain nanoparticle formation four series of copolymers, where the hydrophilic component was MAA and the hydrophobic component was either EHMA, BMA, EMA, or MMA, were synthesized using RAFT copolymerization. The chosen RAFT synthetic approach enabled nearly 100% monomer conversion to be reached and a good control of the molecular weight demonstrating monomodal distribution. The ASCs, initially synthesized in a 50% w/w solution of IPA, produced stable nanoparticle dispersions upon dilution with water and TEA. SAXS analysis has confirmed that SCNPs were formed for each of the copolymer series.

It was found that when the aggregation number of ASCs forming the nanoparticles is close to unity the particle shape transforms from spherical to elongated ellipsoid. For the same ratio of hydrophilic and hydrophobic components, the particle elongation increases with the copolymer molecular weight. The observed dependence can be logically justified based on the minimization of unfavorable interactions of each of the components in the formulation. In an aqueous dispersion of amphiphilic copolymer particles, the hydrophilic groups locate preferentially on the particle surface to be in contact with the aqueous phase, whereas the hydrophobic groups will locate preferentially in the core of the particle. Elongating the particle results in a larger particle surface area to volume ratio, allowing more hydrophilic groups to be located on the particle surface rather than being trapped within the particle core.

The ASC particle morphology analysis demonstrates that two main factors, 1) acidic content associated with the hydrophilic component of the copolymer and 2) molecular weight, control the SCNP formation and elongation from the spherical shape. It was found that for ASCs composed of hydrophobic components with low partition coefficient, SCNPs are formed when the molecular volume is close to the critical spherical particle volume, which is dictated by the ASC composition and can be estimated using the PSC model. Upon a further increase of the ASC molecular weight, when the copolymer exceeds the critical spherical particle volume, the SCNP elongation takes place. This result highlights the main molecular characteristics, namely molecular weight and composition, responsible for the SCNP formation and provides valuable insight into how specific particle morphology can be targeted. Furthermore, the behaviors and dependencies observed for these relatively simple ASC systems containing relatively weak hydrophobic components have the potential to further the understanding of acid group content in more complex and intricate self‐assemblies found in the natural world. Nevertheless, the ASCs composed of hydrophobic comonomers with high partition coefficients demonstrate that particle elongation can take place when the molecular volume is below the critical spherical particle volume when the SCNPs cannot be formed yet. The “early‐elongation” observed for the ASCs containing highly hydrophobic components could be caused by the high MAA content present in these copolymers. However, these additional factors influencing the particle shape formation require further investigations.

## Conflict of Interest

The authors declare no conflict of interest.

## Supporting information



Supporting Information

## Data Availability

The data that support the findings of this study are available in the Supporting Information of this article.
