# Supplementary material for: The Intramolecular Self‐Assembly of Statistical Copolymers in Aqueous Solution to Form Anisotropic Single‐Chain Nanoparticles with Tunable Aspect Ratio
Source: Macromol Rapid Commun. 2024 Dec 27;46(5):2400898. doi: 10.1002/marc.202400898 (PMC11884217; doi:10.1002/marc.202400898)
Supplement: Supplementary file 1 — Supporting Information [file MARC-46-2400898-s001.pdf]

Macromolecular Rapid Communications

# Supporting Information

for *Macromol. Rapid Commun.*, DOI 10.1002/marc.202400898

## The Intramolecular Self-Assembly of Statistical Copolymers in Aqueous Solution to Form Anisotropic Single-Chain Nanoparticles with Tunable Aspect Ratio

*Thomas J. Neal, Rebecca E. Stone, Csilla György, Svetomir B. Tzokov, Sebastian G. Spain* and Oleksandr O. Mykhaylyk**

# Supplementary Information

## The intramolecular self-assembly of statistical copolymers in aqueous solution to form anisotropic single-chain nanoparticles with tunable aspect ratio

Thomas J. Neal,[1] Rebecca E. Stone,[2] Csilla György,[1] Svetomir B. Tzokov,[3] Sebastian G. Spain[1,*] and Oleksandr O. Mykhaylyk[1,*]

[1] *School of Mathematical and Physical Sciences, University of Sheffield, Dainton Building, Sheffield,*

*S3 7HF, UK*

[2] *ICON plc, 500 South Oak Way, Green Park, Reading, RG2 6AG, UK*

[3] *School of Biosciences, University of Sheffield, Firth Court, Western Bank, Sheffield, S10 2TN, UK*

# Contents

Experimental ........ 3
Materials ........ 3
$^1$H Nuclear Magnetic Resonance (NMR) Spectroscopy ........ 3
Gel Permeation Chromatography (GPC) ........ 3
Small-Angle X-ray Scattering measurements ........ 4
Cryo-EM imaging ........ 5
Synthesis of statistical copolymers ........ 5
Supplementary figures ........ 7
Polymerisation Kinetics ........ 7
Molecular weight analysis ........ 9
SAXS and cryo-EM analysis ........ 10
SAXS models ........ 15
Ellipsoid Core-Shell model ........ 15
Gaussian distribution ........ 17
Normalisation by number density per unit volume ........ 17
Linear background ........ 18
Hayter-Penfold structure factor ........ 18
Calculation of the ellipsoidal particle surface area ........ 19
Particle surface charge (PSC) model ........ 19
References ........ 23

# Experimental

## Materials

Butyl methacrylate (BMA, 99%), ethyl methacrylate (EMA, 99%) methyl methacrylate (MMA, 99%), and methacrylic acid (MAA, 99.5 %) were purchased from Sigma-Aldrich (Gillingham, UK). 2-Ethylhexyl methacrylate (EHMA, 98%) was purchased from Alfa Aesar (Heysham, UK). Isopropanol (IPA, 99.9%), triethanolamine (TEA, 99%), 4,4'-azobis(4-cyanovaleric acid) (ACVA), trimethylsilyldiazomethane solution (2.0 M in diethyl ether), and deuterated acetone were purchased from Sigma-Aldrich. Deuterated chloroform and deuterated dimethyl sulfoxide (d6-DMSO) were purchased from VWR (Lutterworth, UK). Deionised water was obtained using an Elgastat Option 3A water purifier (Elga, High Wycombe, UK). 4-((((2-carboxyethyl)thio)carbonothioyl)thio)-4-cyanopentanoic acid (CECPA) used for RAFT polymerization was supplied by Boron Molecular (Raleigh, NC, USA). Unless stated otherwise, all materials were used as received.

## $^1$H Nuclear Magnetic Resonance (NMR) Spectroscopy

$^1$H NMR spectra were recorded in either $d_6$-DMSO, d6-acetone or $CDCl_3$ using a Bruker AV1-400 or AV3HD-400 MHz spectrometer. These spectra were analyzed using Bruker Topspin 3.5pl7 software and chemical shifts are reported relative to a residual solvent peak.

## Gel Permeation Chromatography (GPC)

Molecular weight distributions of the statistical copolymers were determined by GPC using THF containing 4% v/v acetic acid and 0.025% w/v butyl hydroxytoluene (BHT)

as the eluent. Due to a high level of acid present copolymers composed of EHMA, their MAA units were esterified with trimethylsilyl (TMS)-diazomethane prior to the measurements. A PL-GPC50 integrated GPC system (Agilent, UK) equipped with a refractive index detector was used for the analysis. Separations were carried out using a pair of PLgel Mixed-C columns (7.8 × 300 mm, 5 μm bead size), fitted with a PLgel guard column (7.8 × 50 mm, 5 μm bead size), at a flow rate of 1.0 mL min$^{-1}$. All the samples were measured relative to a set of ten low-dispersity PMMA standards (Agilent, UK) with peak molecular weight values ranging from 550 Da to 1,568,000 Da.

## Small-Angle X-ray Scattering measurements

Throughout this work SAXS patterns were collected using laboratory SAXS instruments [either a Nanostar (Bruker AXS, Germany) equipped with a two-dimensional (2D) Hi-STAR multi-wire gas detector and modified with GeniX 3D X-ray source (Cu$K_\alpha$ radiation, X-ray wavelength $\lambda$ = 1.54 Å) and motorised collimating scatterless slits (Xenocs, France) or Xeuss 2.0 laboratory beamline (Xenocs, France) equipped with a 2D Pilatus 1M detector (Dectris, Switzerland) and a liquid gallium MetalJet X-ray source (Excillum, Sweden) ($\lambda$ = 1.34 Å)]. Glass capillaries with a diameter of 2 mm (WJM-Glas, Berlin, Germany) were used as sample holders. The patterns were collected over a scattering vector length range of 0.008 Å$^{-1}$ < $q$ < 0.16 Å$^{-1}$, where $q = \frac{4\pi}{\lambda}\sin\theta$ and $\theta$ is a half of the scattering angle. One-dimensional (1D) scattering curves were obtained by an azimuthal binning and averaging of corresponding two-dimensional scattering patterns using software packages supplied with the SAXS instruments. Calibration to absolute intensity achieved using the SAXS pattern recorded for deionized water (assuming that the differential scattering cross-section of water at 20°C is 0.0165 cm$^{-1}$). Calibration and background subtraction were

performed using Irena SAS macros for Igor Pro[43] and further analysis of the 1D was performed using SAS fit.

## Cryo-EM imaging

Polymer dispersions at a concentration of 0.1% w/w were used for cryo-EM imaging. A 5 μl sample of the dispersions was applied to a glow-discharged copper Quantifoil R2/2 300 mesh holey carbon grid, with an optimized blotting time of 3 seconds, and vitrified by plunging into liquid ethane using a Leica EM GP automatic plunge freezer (Leica Microsystems). Cryo-EM images were acquired using a Tecnai Arctica microscope (ThermoFisher Scientific) operating at 200 kV, with data recorded on a Falcon III direct electron detector (ThermoFisher Scientific). Images were collected with an object-level pixel size of 0.931 Å and a total electron dose of 27 electrons/$Å^2$ or less.

## Synthesis of statistical copolymers

The copolymerization protocol for producing a P(*A*-*stat*-*B*) amphiphilic statistical copolymer, where *A* is hydrophobic comonomer and *B* is hydrophilic comonomer, is illustrated here for all cases by a synthesis of P(EMA-*stat*-MAA) with EMA to MAA molar ratio of 60 to 40 with a degree of polymerization (DP) of 250 ($EM_{250}$, see Table 1). EMA (Monomer *A*, 1.33 g, 11.6 mmol), MAA (Monomer *B*, 0.669 g, 7.78 mmol), ACVA (4.36 mg, 0.016 mmol), and PETTC (26.4 mg, 0.078 mmol) were mixed in IPA (2.03 g) creating a 50% w/w monomer solution, and placed in an ice bath to cool. The mixture was degassed with $N_2$ for 20 min and then heated to 70 °C to initiate the

reaction. The copolymerization was allowed to proceed for 24 h before quenching by cooling to ambient temperature with concomitant exposure to air.

# Supplementary figures

## Polymerisation Kinetics

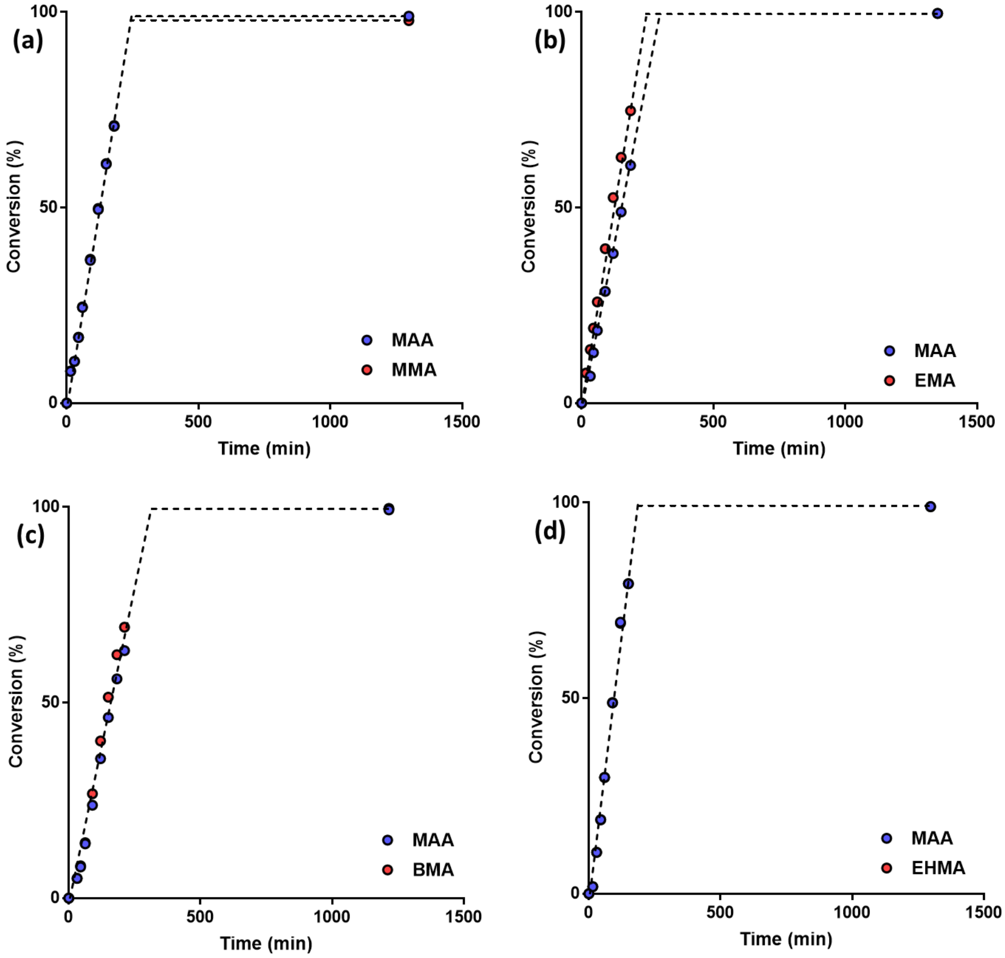


**Figure S1.** Monomer conversion during synthesis of statistical copolymers by RAFT at 50% w/w in IPA as determined by $^{1}H$ NMR spectroscopy: (a) MMA and MAA [70:30], (b) EMA and MAA [60:40], (c) BMA and MAA [50:50], and (d) EHMA and MAA [35:65].

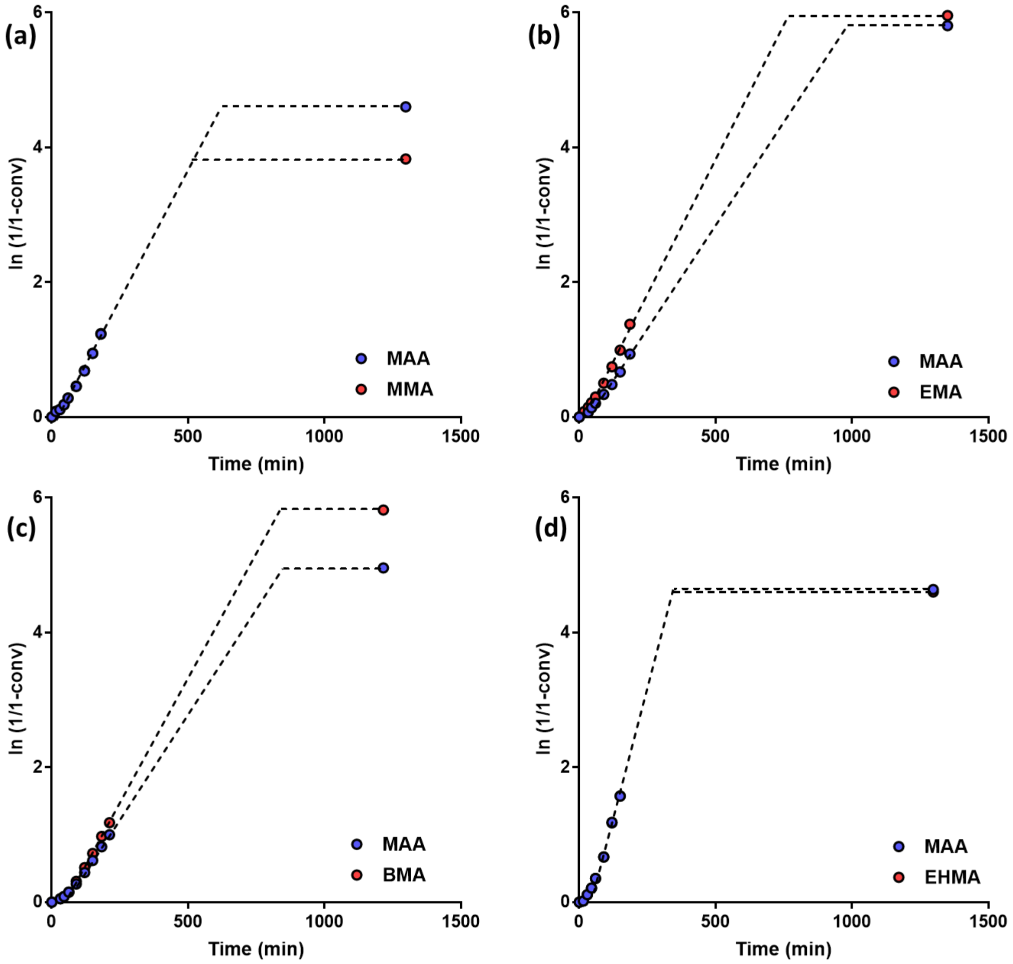


**Figure S2.** Pseudo-first order kinetic plots of monomer conversion against time for (a) MMA and MAA [70:30], (b) EMA and MAA [60:40], (c) BMA and MAA [50:50], and (d) EHMA and MAA [35:65].

## Molecular weight analysis

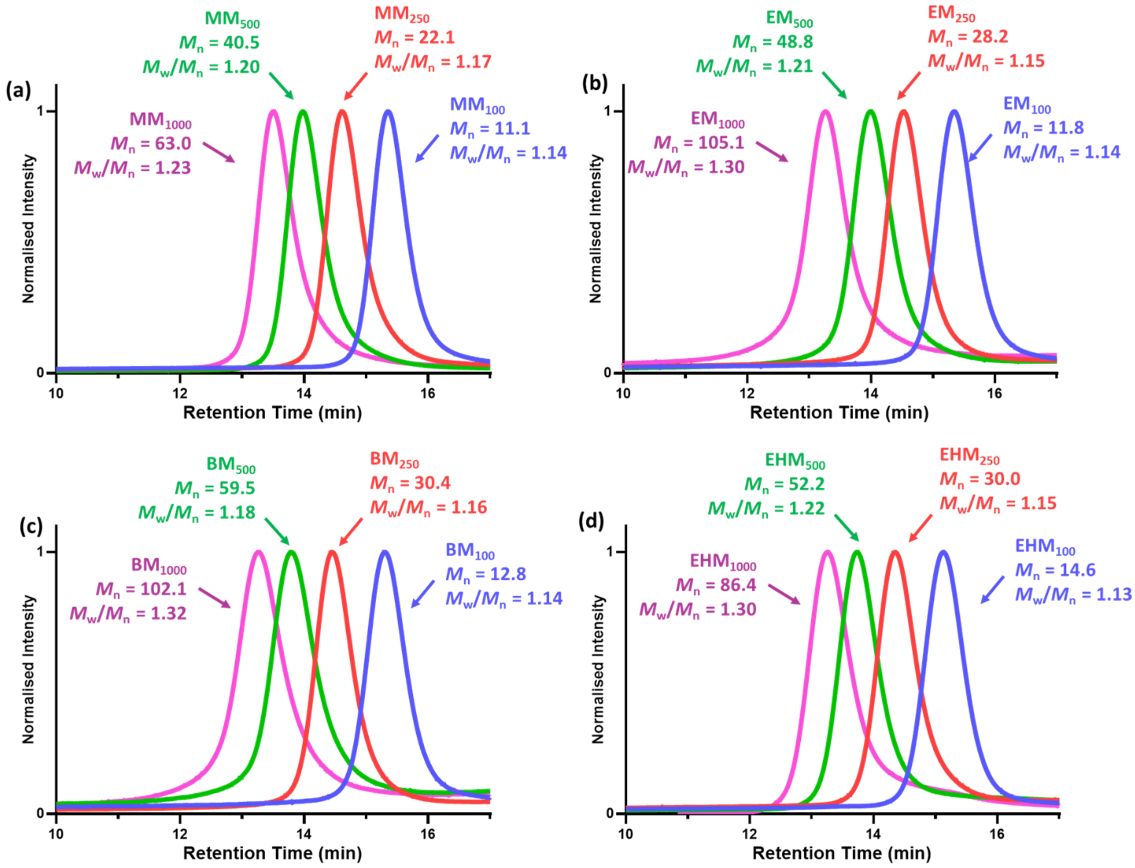


**Figure S3.** GPC chromatograms of the (a) poly(MMA-*stat*-MAA), (b) poly(EMA-*stat*-MAA), (c) poly(BMA-*stat*-MAA), and (d) poly(EHMA-*stat*-MAA) copolymer series synthesised by RAFT solution polymerisation at 50% w/w in IPA. GPC was performed in THF containing 4% v/v acetic acid against PMMA standards. The molecular weight ($M_n$) is given in kDa units.

SAXS and cryo-EM analysis

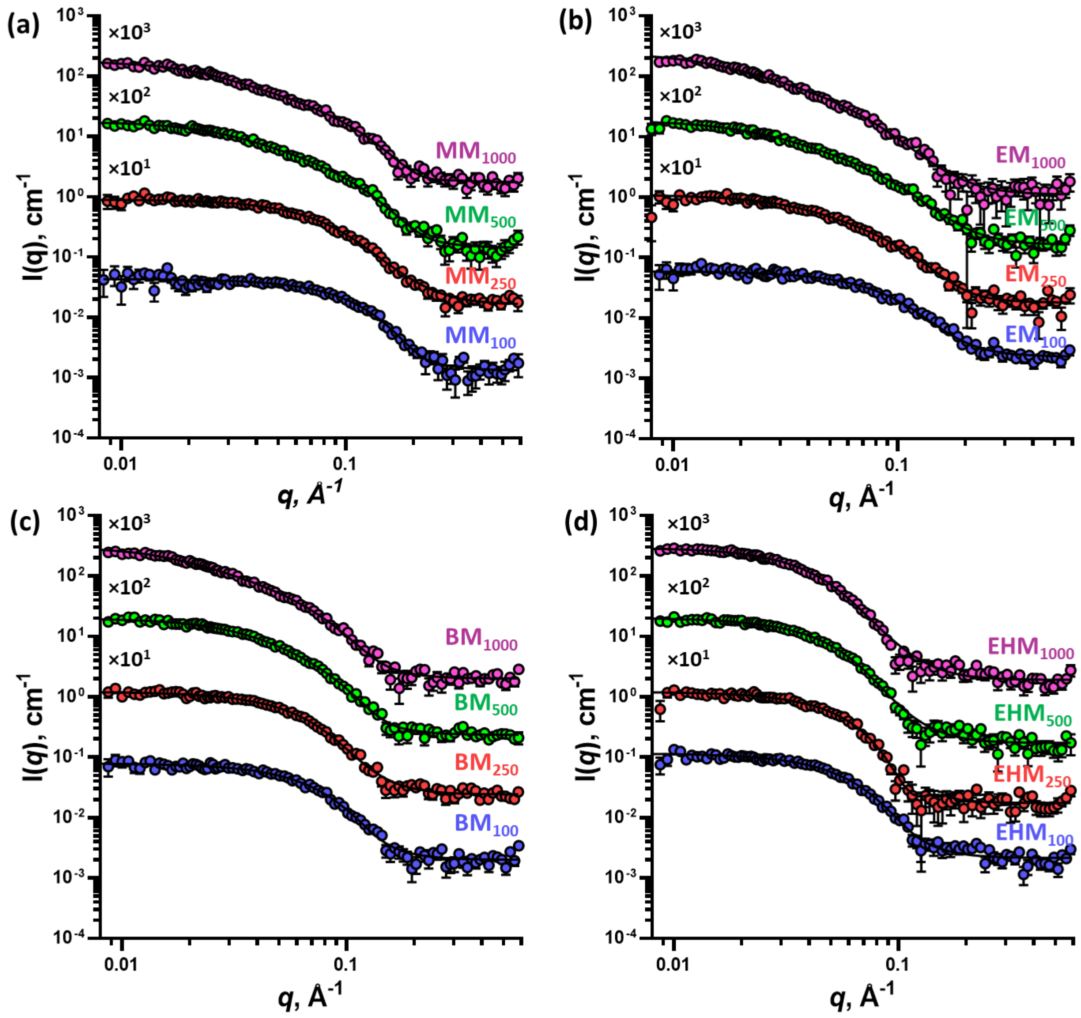


**Figure S4.** SAXS patterns recorded for 1.0% w/w aqueous dispersions of P(*X*MA-*stat*-MAA) copolymer particles with 0.05M of NaCl (symbols) where (a) P(MMA-*stat*-MAA), (b) P(EMA-*stat*-MAA), (c) P(BMA-*stat*-MAA), and (d) P(EHMA-*stat*-MAA) fitted using an ellipsoid core-shell model with a linear background (eqs S2-S8 and S13) (solid lines). A Xenocs Xeuss 2.0 instrument was used for these measurements. Some patterns are shifted upwards by arbitrary numerical factors shown at the top of corresponding patterns to aid clarity.

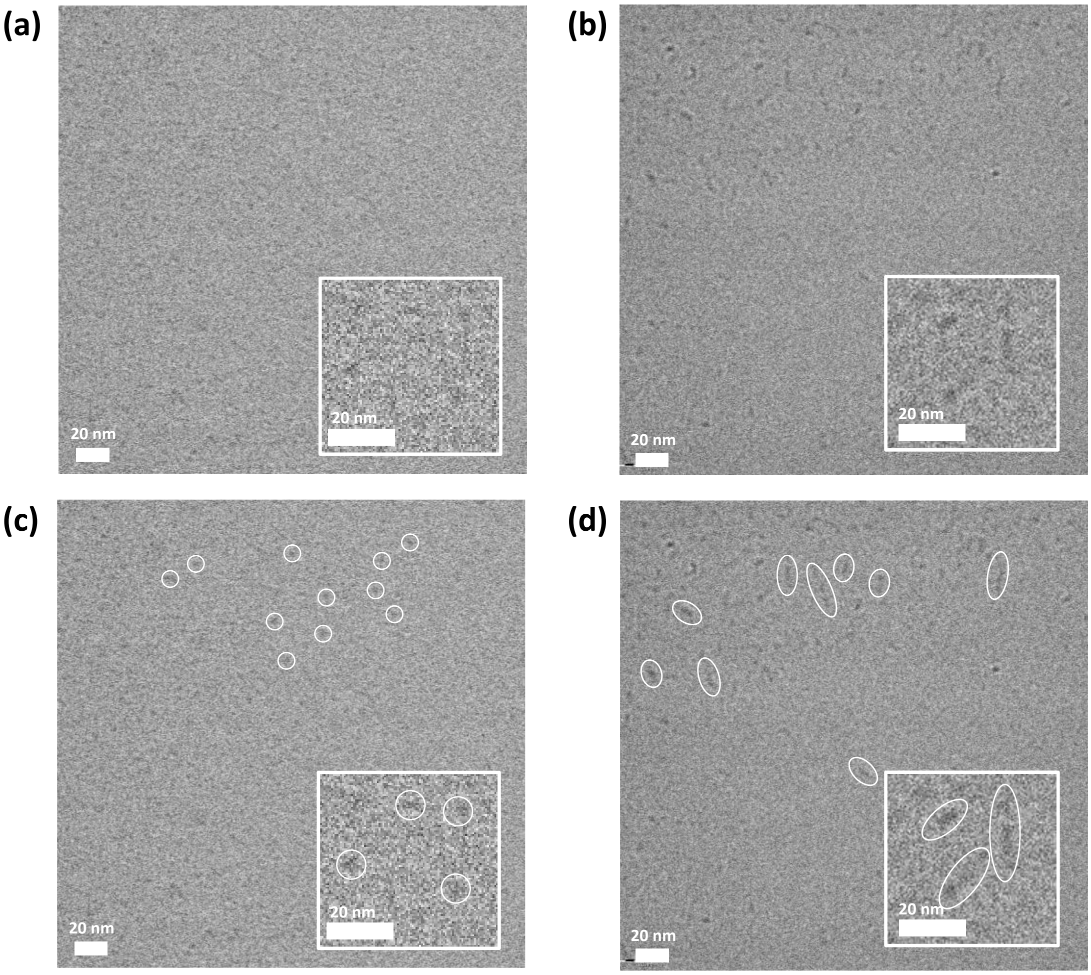


**Figure S5.** Cryo-TEM images recorded for (a) $EHM_{100}$ spherical particles and (b) $EM_{1000}$ elongated particles. (c) and (d) are $EHM_{100}$ and $EM_{1000}$ dispersion, respectively, with the particles highlight using a white outline. The white scale bar in each TEM image corresponds to 20 nm.

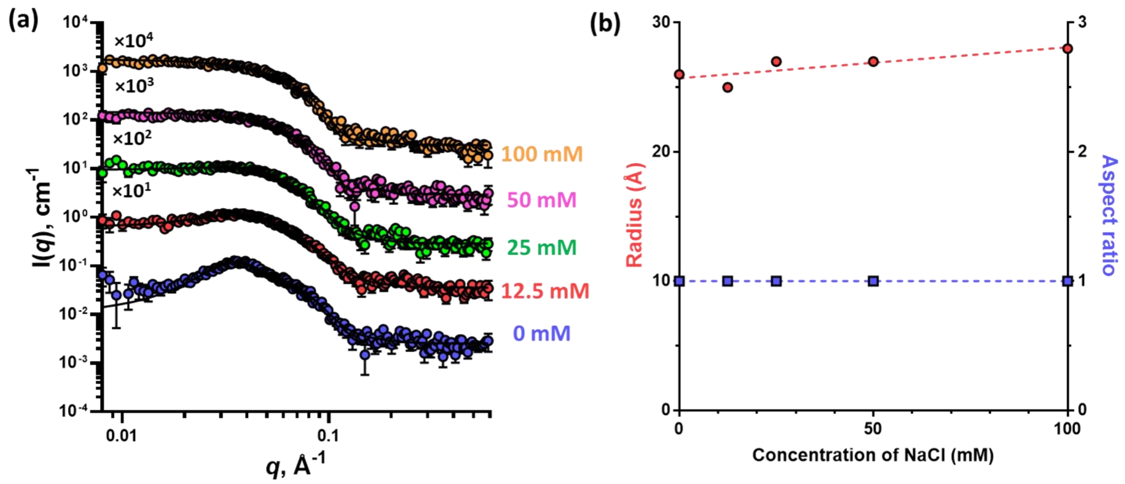


**Figure S6.** (a) SAXS patterns recorded for 1.0% w/w aqueous dispersions of $EHM_{250}$ copolymer particles (symbols) with different concentrations of NaCl. Some patterns are shifted upwards by arbitrary numerical factors shown at the top of corresponding patterns to aid clarity. (b) The polar radius ($R_p$) and aspect ratio ($\varepsilon$) of the particles formed by $BM_{500}$ copolymer at different concentrations of NaCl. The lines are shown for eye guidance. A Xenocs Xeuss 2.0 instrument was used for these measurements.

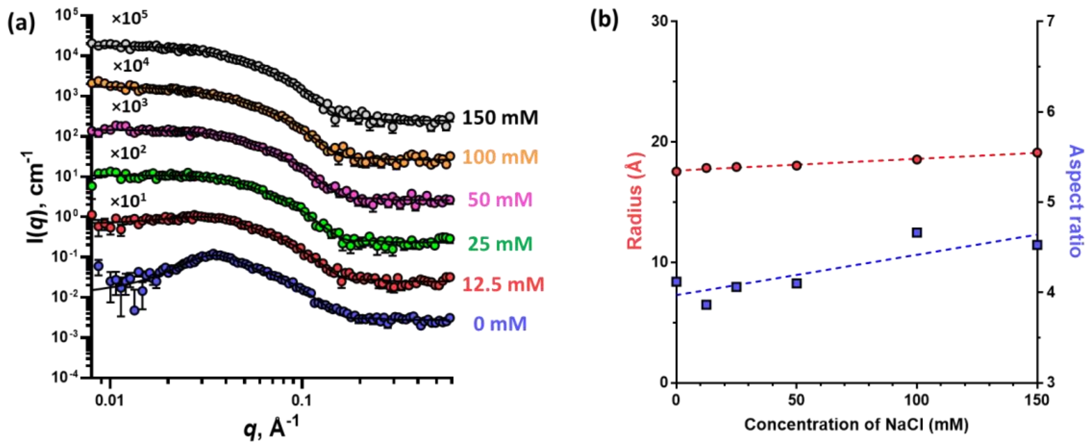


**Figure S7.** (a) SAXS patterns recorded for 1.0% w/w aqueous dispersions of $BM_{500}$ copolymer particles (symbols) with varying levels of NaCl salt added. Some patterns are shifted upwards by arbitrary numerical factors shown at the top of corresponding patterns to aid clarity. (b) The polar radius ($R_p$) and aspect ratio ($\varepsilon$) of the particles formed by $BM_{500}$ copolymer at different NaCl salt concentrations. The lines are shown for eye guidance. A Xenocs Xeuss 2.0 instrument was used for these measurements.

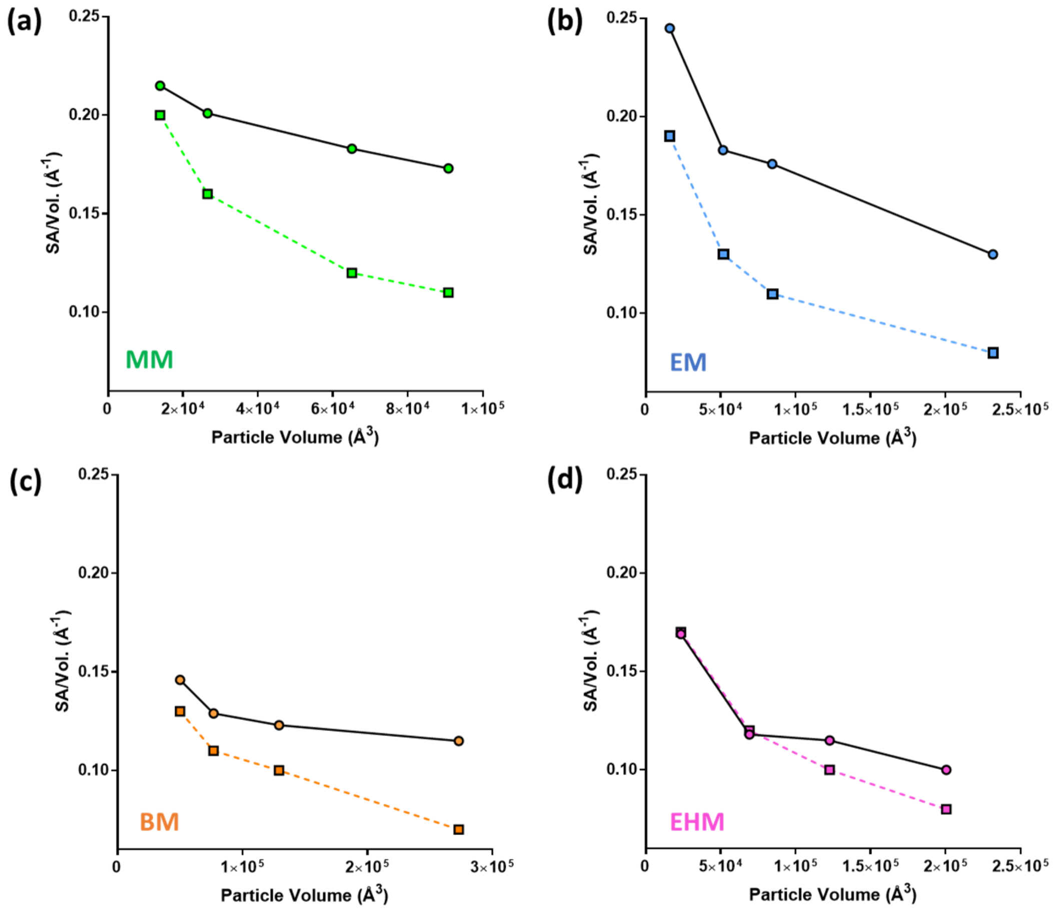


**Figure S8.** The particle surface area per particle volume (SA/$V_c$) against the particle volume ($V_c$) for the ellipsoid particles calculated from SAXS results (circles) and the hypothetical spherical particles calculated based upon the known volumes of the ellipsoid particles (squares) where (a) is the MM series, (b) is the EM series, (c) is the BM series, and (d) is the EHM series. The lines are shown for eye guidance.

# SAXS models

The intensity of the X-rays scattered by a particle dispersion, $I(q)$ is defined as the product of the particle form factor and the structure factor:

$$I(q) = NS(q)\int_0^\infty \dots \int_0^\infty F\,(q, r_1, \dots, r_k)\Psi(r_1, \dots, r_k)dr_1 \dots dr_k \quad \text{(S1)}$$

where $F(q, r_1, \dots, r_k)$ is the form factor defined by a $k$ number of $r$ parameters, $\Psi(r_1, \dots, r_k)$ is the distribution function of these parameters, $N$ is the number density per unit volume, and $S(q)$ is the structure factor.

## Ellipsoid Core-Shell model

An ellipsoid core-shell model used in this paper to model particles that are elongated,[1,2] whilst taking into account the associated ion shell that surrounds the charged particle (Figure S8). The ionic shell is caused by the associated base triethanol amine (TEA) which is highly scattering with respect to the background solvent and thus must be considered in the model.[3]

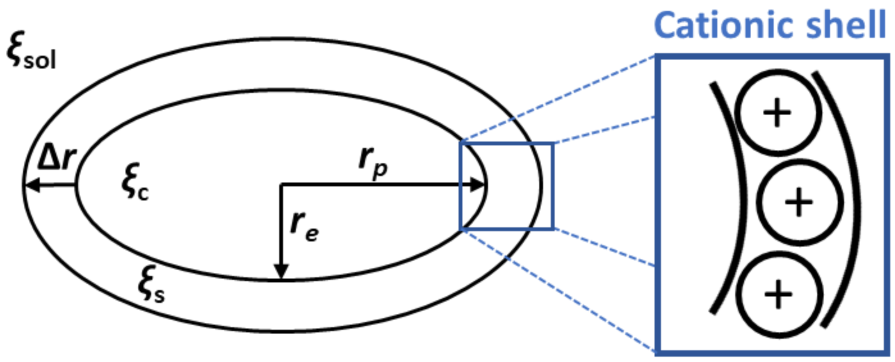


**Figure S9**. A schematic of the ellipsoid core-shell model corresponding to a prolate ellipsoid, where $r_p$ is the polar core radius, $r_e$ is the equatorial core radius, $\Delta r$ is the ionic shell thickness (6 Å), $\xi_c$ is the SLD of the particle core, $\xi_s$ is the SLD of the associated ion shell, and $\xi_{sol}$ is the SLD of the solvent.

The form factor is defined as:

$$F_{\mathrm{E-cs}}(q, r_{\mathrm{p}}, r_{\mathrm{e}}, \Delta r) = \int_0^1 A_{\mathrm{E-cs}}{}^2(q, r_{\mathrm{p}}, r_{\mathrm{e}}, \Delta r, \mu)\, d\mu \quad \text{(S2)}$$

In this case the form factor amplitude contains information about the scattering length densities of the particle and the solvent background and is defined as:

$$A_{\mathrm{S-cs}}(q, r_{\mathrm{p}}, r_{\mathrm{e}}, \Delta r, \mu) = (\xi_{\mathrm{c}} - \xi_{\mathrm{sol}})V_{\mathrm{c}}\left[\frac{3f(qx_{\mathrm{c}})}{x_{\mathrm{c}}}\right] + (\xi_{\mathrm{s}} - \xi_{\mathrm{sol}})V_{\mathrm{t}}\left[\frac{3f(qx_{\mathrm{s}})}{x_{\mathrm{s}}}\right] \quad \text{(S3)}$$

where $r_{\mathrm{p}}$ and $r_{\mathrm{e}}$ are the polar and equatorial radius of the particle core, respectively, related to each other via the aspect ratio parameter $\varepsilon = r_{\mathrm{p}}/r_{\mathrm{e}}$. $\Delta r$ is the ion shell thickness and was fixed at the approximate length of a single TEA unit (6 Å ).[3] $\xi_{\mathrm{sol}}$, $\xi_{\mathrm{s}}$ and $\xi_{\mathrm{c}}$ are the SLDs of the solvent, the ion shell and the particle core, respectively. $\xi_{\mathrm{c}}$ in this case corresponds to the averaged scattering length density of the copolymer calculated as:

$$\xi_{\mathrm{c}} = \xi_x \cdot v_x + \xi_y \cdot v_y \quad \text{(S4)}$$

where $\xi_x$ and $\xi_y$ are the scattering length densities of $x$ and $y$ copolymer repeat units, and $v_x$ and $v_y$ are the volume fractions of $x$ and $y$ units, respectively. $f(z)$ is the scattering amplitude of an ellipsoid defined as:

$$f(z) = \frac{\sin(z) - z\cos(z)}{z^2} \quad \text{(S5)}$$

The size parameters in eq S3 are expressed as:

$$x_{\mathrm{c}} = \sqrt{r_{\mathrm{p}}{}^2\mu^2 + r_{\mathrm{e}}{}^2(1-\mu^2)} \quad \text{(S6)}$$

$$x_{\mathrm{s}} = \sqrt{(r_{\mathrm{p}} + \Delta r)^2\mu^2 + (r_{\mathrm{e}} + \Delta r)^2(1-\mu^2)} \quad \text{(S7)}$$

$V_{\mathrm{c}}$ and $V_{\mathrm{t}}$ are the particle core and the total particle volume, respectively:

$$V_{\mathrm{c}} = \frac{4}{3}\pi r_{\mathrm{p}} r_{\mathrm{e}}{}^{2} \quad \text{(S8)}$$

$$V_{\mathrm{t}} = \frac{4}{3}\pi\left(r_{\mathrm{p}} + \Delta r\right)\left(r_{\mathrm{e}} + \Delta r\right)^{2} \quad \text{(S9)}$$

Additionally, the aggregation number ($N_{agg}$) can be calculated as:

$$N_{\mathrm{agg}} = \frac{4\pi r_{\mathrm{e}}^{2} r_{\mathrm{p}}}{3V_{\mathrm{poly}}} \quad (S10)$$

where $V_{poly}$, is the calculated volume of a single copolymer molecule using the equation:

$$V_{\mathrm{poly}} = \left(\frac{M_{\mathrm{n}x}}{\rho_{x} \times N_{\mathrm{A}}}\right) + \left(\frac{M_{\mathrm{n}y}}{\rho_{y} \times N_{\mathrm{A}}}\right) \quad \text{(S11)}$$

where $M_{nx}$ and $M_{ny}$ are the molecular weights of the polymer components $x$ and $y$, respectively, $\rho_x$ and $\rho_y$ are the mass densities of the individual polymer components $x$ and $y$, respectively (where the densities of PMMA, PEMA, PBMA, PEHMA and PMAA are 1.20 g $cm^{-3}$, 1.11 g $cm^{-3}$, 1.05 g $cm^{-3}$, 0.96 g $cm^{-3}$, and 1.25 g $cm^{-3}$, respectively) and $N_A$ is Avogadro's number.

The ellipsoid core-shell model is equivalent to a spherical core-shell model when $r_e$ = $r_p$ (Figure S9).

## Gaussian distribution

Only polydispersity of the particle equatorial radius, expressed as a Gaussian distribution, is considered for the structural model (eq S1):

$$\Psi(r_{\mathrm{e}}) = \frac{1}{\sqrt{2\pi\sigma_{R_{\mathrm{e}}}^{2}}} e^{-\frac{(r_{\mathrm{e}} - R_{\mathrm{e}})^{2}}{2\sigma_{R_{\mathrm{e}}}^{2}}} \quad \text{(S12)}$$

where $R_e$ is the mean particle equatorial radius and $\sigma_{Re}$ is its standard deviation.

## Normalisation by number density per unit volume

The number density per unit volume used in eq S1 is expressed as:

$$N = \frac{\phi}{\int_0^\infty V_c(r)\Psi(r)dr} \quad \text{(S13)}$$

where $\phi$ is the total volume fraction of copolymer particles in the sample and $V_c(r)$ is the core *volume* of the copolymer particle (eq S8).

## Linear background

A plateau in intensity at high $q$ is observed in the majority of the scattering patterns collected. This plateau is likely caused by fluctuations in scattering length density across the particle because of different chemical composition of the repeat units composing the statistical copolymers. In order to account for these fluctuations a linear fitting parameter ($C_1$) that is independent of the scattering vector $q$ is incorporated into the structural model (eq S1):

$$I(q) = NS(q) \int_0^\infty \dots \int_0^\infty F\,(q, r_1, \dots, r_k)\Psi(r_1, \dots, r_k)dr_1 \dots dr_k + C_1 \quad \text{(S14)}$$

## Hayter-Penfold structure factor

A charged particle Coulomb interaction is usually described by the Hayter-Penfold structure factor:[4]

$$S(q) = S_{\mathrm{HP}}(q, R_{\mathrm{HP}}, f_{\mathrm{HP}}, M, T, \varepsilon_{\mathrm{d}}, Q) \quad \text{(S15)}$$

where $R_{\mathrm{HP}}$ is an interparticle correlation radius (half of the interparticle correlation distance), $f_{\mathrm{HP}}$ is an effective volume fraction, $M$ is the ionic strength of the solvent, $T$ is the absolute temperature, $\varepsilon_{\mathrm{d}}$ is the solvent dielectric constant and $Q$ is the particle charge expressed in electrons. $R_{\mathrm{HP}}$ and $Q$ were fit parameters, whereas the parameters were fixed during fitting with the following values: $f_{\mathrm{HP}} = 0.15$, $M = 0$, $T = 298$ K and $\varepsilon_{\mathrm{d}} = 78$ (dielectric constant of water at 298 K).

In the case where the charge was shielded by the addition of salt, no structure factor was observed and thus equation S15 becomes $S(q) = 1$.

## Calculation of the ellipsoidal particle surface area

The surface area of an ellipsoid cannot be expressed exactly by an elementary function. However, an approximate formula is often used:

$$SA = 4\pi \left[ \frac{2\left(r_{\mathrm{p}}^{p} r_{\mathrm{e}}^{p}\right) + 2 r_{\mathrm{e}}^{p}}{3} \right]^{1/p} \qquad \text{(S16)}$$

where $p \approx 1.61$.

## Particle surface charge (PSC) model

The PSC model is used to describe the relationship between the particle (in this case the core) radius ($R$) and copolymer composition for statistical copolymers based on the assumption that a critical fractional surface coverage ($SA_{\mathrm{frac}}$) by charged ($x$) units is required for colloidal stability:[3,5]

$$N_{x,\mathrm{p}} = \frac{1}{k} \times \frac{4\pi R^2 \times SA_{\mathrm{frac}}}{CS_x} \qquad \text{(S17)}$$

where $N_{x,\mathrm{p}}$ is the mean number of charged $x$ groups per nanoparticle, $CS_x$ is the cross-sectional area of a single $x$ repeat unit calculated from the approximate volume of a single $x$ unit, $V_x$, ($CS_x = V_x^{2/3}$) and $k$ is the fraction of the $x$ groups located at the particle surface. $k$ equals 1 when all the anionic $B$ groups are located at the nanoparticle surface. Alternatively, if all the $x$ units are buried within the nanoparticle cores, $k$ equals 0. In the latter case the nanoparticles are not colloidally stable ($N_{x,\mathrm{p}}$ tends to infinity, suggesting that an infinitely large particle would be required to form a stable dispersion). Previous small angle neutron scattering has determined $k$ to be about 0.5. Following eq S17, the mean number of hydrophobic $y$ groups per nanoparticle ($N_{y,\mathrm{p}}$) can be expressed as:

$$N_{y,\mathrm{p}} = \frac{\frac{4}{3}\pi R^3 - \left(\frac{1}{k} \times \frac{4\pi R^2 \times SA_{\mathrm{frac}} \times V_x}{CS_x}\right)}{V_y} \qquad (S18)$$

where $V_y$ is the volume of a single hydrophobic unit.

Using both $N_{x,\mathrm{p}}$ and $N_{y,\mathrm{p}}$, the mole fraction of $x$ groups in a nanoparticle can be calculated as:

$$Mole\ fraction\ of\ x = \frac{N_{x,\mathrm{p}}}{N_{x,\mathrm{p}} + N_{y,\mathrm{p}}} \qquad (S19)$$

Previously, the $SA_{\mathrm{frac}}$ for a set of P(*X*MA-*st*-MAA) copolymers was determined (where *X*MA was either MMA, EMA, BMA and EHMA).[3,5] Thus, using the described relationships (eqs S16 – S18) the radius of copolymer particles at specific compositions can be determined (Table S1).

**Table S1.** Particle characteristics (particle radius, *R*, and critical fractional surface coverage by hydrophilic units, $SA_{\mathrm{frac}}$) and copolymer properties (molecular volume, $V_{\mathrm{poly}}$, degree of polymerisation, DP, and molecular weight, $M_{\mathrm{n}}$) required to achieve single chain nanoparticles for a given copolymer composition (molar fractions of hydrophobic units, *X*MA mol frac, and hydrophilic units, MAA mol frac) and hydrophobicity predicted using the PSC model.

| copolymer | $SA_{\mathrm{frac}}$ | *X*MA mol frac | MAA mol frac | *R* (Å) | $V_{\mathrm{poly}}$ (Å$^3$) | DP | $M_{\mathrm{n}}$ (kDa) |
|---|---|---|---|---|---|---|---|
| MM | 0.07 | 0.7 | 0.3 | 7.62 | 1850 | 14 | 1.3 |
| EM | 0.13 | 0.6 | 0.4 | 11.55 | 6460 | 43 | 4.4 |
| BM | 0.23 | 0.5 | 0.5 | 19.46 | 30900 | 179 | 20.4 |
| EHM | 0.50 | 0.35 | 0.65 | 37.45 | 220000 | 1108 | 138.7 |

Assuming that the size of the particles formed by these statistical copolymers is independent of molecular weight, as has been previously reported,[5] the volume ($V_{\text{poly}}$), molecular weight ($M_{\text{n}}$) and degree of polymerisation (DP) of a copolymer molecule which could potentially form a single chain nanoparticle ($N_{\text{agg}}$ = 1) can be calculated (Table S1):

$$V_{\text{poly}} = \frac{4}{3}\pi R^3 \quad \text{(S20)}$$

$$\text{DP} = \frac{V_{\text{poly}}}{\left(\chi_x V_x + \chi_y V_y\right)} \quad \text{(S21)}$$

$$M_{\text{n}} = \text{DP} \times \left(\chi_x M_{\text{w}x} + \chi_y M_{\text{w}y}\right) \quad (S22)$$

where, $V_{\text{poly}}$ is the volume of the polymer, $\chi_x$ and $\chi_y$ are the mole fractions of monomers *x* and *y*, respectively, $V_x$ and $V_y$ are the volumes of the hydrophilic monomer repeat units (MAA = 121.08 Å$^3$) and the hydrophobic monomer repeat units (MMA = 138.38 Å$^3$, EMA = 170.54 Å$^3$, BMA = 224.00 Å$^3$, and EHMA = 342.49 Å$^3$), respectively, and $M_{\text{w}x}$ and $M_{\text{w}y}$ are the molecular weights of the *x* and *y* repeat units, respectively.

**Table S2.** Scattering length densities using to model the SAXS patterns.

| *X*MA | Copolymer name | Targeted Composition (*X*MA:MAA) | SLD × $10^{-10}$ $cm^{-2}$ |
|---|---|---|---|
| MMA | $MM_{100}$ | 70:30 | 11.005 |
| | $MM_{250}$ | 70:30 | 11.005 |
| | $MM_{500}$ | 70:30 | 11.005 |
| | $MM_{1000}$ | 70:30 | 11.005 |
| EMA | $EM_{100}$ | 60:40 | 10.609 |
| | $EM_{250}$ | 60:40 | 10.609 |
| | $EM_{500}$ | 60:40 | 10.609 |
| | $EM_{1000}$ | 60:40 | 10.609 |
| BMA | $BM_{100}$ | 50:50 | 10.308 |
| | $BM_{250}$ | 50:50 | 10.308 |
| | $BM_{500}$ | 50:50 | 10.308 |
| | $BM_{1000}$ | 50:50 | 10.308 |
| EHMA | $EHM_{100}$ | 35:65 | 9.608 |
| | $EHM_{250}$ | 35:65 | 9.608 |
| | $EHM_{500}$ | 35:65 | 9.608 |
| | $EHM_{1000}$ | 35:65 | 9.608 |
| Solvent | H2O | | 9.420 |
| | TEA | | 9.980 |
| | NaCl(aq) 50mM | | 9.431 |

# References

1. Kotlarchyk, M. & Chen, S. H. Analysis of small angle neutron scattering spectra from polydisperse interacting colloids. *J. Chem. Phys.* **79**, 2461–2469 (1983).

2. Berr, S. Solvent Isotope Effects on Alkyltrimethylammonium Bromide Micelles as a Function of Alkyl Chain Length. *J. Phys. Chem* **91**, 4760 (1987).

3. Neal, T. J. *et al.* Control of Particle Size in the Self-Assembly of Amphiphilic Statistical Copolymers. *Macromolecules* **54**, 1425–1440 (2021).

4. Hayter, J. B. & Penfold, J. An analytic structure factor for macroion solutions. *Mol. Phys.* **42**, 109–118 (1981).

5. Neal, T. J. *et al.* Self-Assembly of Amphiphilic Statistical Copolymers and Their Aqueous Rheological Properties. *Macromolecules* **51**, 1474–1487 (2018).
